# Interleukin 27 Induces the Expression of Complement Factor H (CFH) in the Retina

**DOI:** 10.1371/journal.pone.0045801

**Published:** 2012-09-20

**Authors:** Ahjoku Amadi-Obi, Cheng-Rong Yu, Ivy Dambuza, Sung-Hye Kim, Bernadette Marrero, Charles E. Egwuagu

**Affiliations:** Molecular Immunology Section, National Eye Institute, National Institutes of Health, Bethesda, Maryland, United States of America; The Ohio State University, United States of America

## Abstract

Complement factor H (CFH) is a central regulator of the complement system and has been implicated in the etiology of age-related macular degeneration (AMD), a leading cause of blindness in the elderly. In view of previous studies showing that reduced expression of CFH in the retina is a risk factor for developing AMD, there is significant interest in understanding how CFH expression is regulated in the retina. In this study, we have shown that the anti-inflammatory cytokine, IL-27, induced CFH expression in mouse retinal cells and human retinal pigmented epithelial cells (RPE) through STAT1-mediated up-regulation of Interferon Regulatory Factor-1 (IRF-1) and IRF-8. We further show that cells in the ganglion and inner-nuclear layers of the retina constitutively express IRF-1 and IRF-8 and enhanced CFH expression in the retina during ocular inflammation correlated with significant increase in the expression of IRF-1, IRF-8 and IL-27 (IL-27p28 and Ebi3). Our data thus reveal a novel role of IL-27 in regulating complement activation through up-regulation of CFH and suggest that defects in IL-27 signaling or expression may contribute to the reduction of CFH expression in the retina of patients with AMD.

## Introduction

The vertebrate eye is comprised of highly specialized tissues of the retina, cornea, lens, iris, ciliary body, choroid, sclera, aqueous humor and vitreous body and its primary function is to receive and convert incident light rays into visual images. To ensure the fidelity of the visual image, the optical axis (cornea, anterior chamber, lens and vitreous body) through which the light is transmitted, must be transparent and devoid of lymphoreticular cells [1]. Intraocular inflammation, characterized by influx of inflammatory cells into the retina, is therefore highly undesirable because it compromises the requisite transparency of the optical axis. Moreover, inflammatory mediators secreted by the inflammatory cells promote undesirable angiogenesis, fibrosis and tissue destruction [1]. Thus, ocular immunity is predicated on the paramount need to preserve vision while neutralizing pathogenic stimuli with minimal collateral damage to ocular structures. The complement system plays a central role in conferring innate immunity against microbes in the eye and it functions through sequential activation of cascades of proteases and a cell-killing membrane attack complex that generate cleavage fragments which amplify the inflammatory response. The uncontrolled activation of the complement pathway in the eye is therefore dangerous as it is potentially injurious to terminally differentiated photoreceptors and neurons of the neuroretina. This calamity is obviated by the production of a family of complement control proteins of which complement factor H (CFH) is a prominent member.

CFH is a multifunctional protein that acts as a central regulator of the complement system [2]. It regulates the levels of newly generated C3b molecules by serving as a cofactor for complement factor I in degrading C3b [2] and destabilizing the alternative complement C3 convertase, C3bBb (decay-accelerating activity). *CFH* is one of several genes identified in genome-wide association studies [3], [4], [5], [6], [7], [8] to be implicated in age-related macular degeneration (AMD), the leading cause of irreversible blindness in the elderly [9], [10], [11]. Although the pathophysiology of AMD is not well understood, recent reports suggest that defects in mechanisms that control chronic inflammation or down-regulate complement activation play crucial roles [9], [10], [11]. *CFH* variants that express reduced amounts of CFH or have low complement-modulating activity have been suggested as risk factors for developing AMD [12].

CFH is constitutively expressed in the liver and the liver produces the highest level of CFH in the body [2]. Significant amounts of CFH are also produced in the eye, with the highest levels detected in the retinal pigment epithelia (RPE), choroid and the retina [13]. The pro-inflammatory cytokine, IFN-g has been shown to up-regulate the expression of CFH in hepatocytes, astrocytes, lung cells and astroglioma cell lines [14], [15] and these observations are consistent with the presence of two IFN-g activation sites (GAS) within the *CFH* proximal promoter [16], [17], [18]. With the knowledge that defects in CFH production may contribute to the development of AMD, it is remarkable that less is known about mechanisms involved in the regulation of CFH production in the normal retina. The transcription factor, STAT1 (signal transducer and activator of transcription 1), has been shown to activate the transcription of *CFH* in the human retinal pigment epithelia (RPE) cell line, ARPE-19 following stimulation by IFN-g [12], [19]. On the other hand, oxidative stress reduced the ability of IFN-g to increase CFH expression in ARPE-19 by enhancing FOXO3a binding to the CFH promoter and inhibiting interaction of STAT1 with the CFH promoter [12], [19]. These studies established the central role of STAT1 in the regulation of CFH in RPE. However, IFN-g is not present in the normal retina nor is it secreted by retinal cells and could therefore not be responsible for activating STAT1 required for constitutive expression of CFH in the retina. We therefore posited that other cytokines such as IL-27 or growth factors produced by retinal cells may be the source of the activated STAT1 needed to induce transcription of the *CFH* gene.

Interleukin-27 (IL-27) is an anti-inflammatory heterodimeric cytokine comprised of IL-27p40 and EBI3 (Epstein-Barr virus (EBV)-induced gene 3) subunits [20]. It is constitutively secreted by retinal microglia and in a previous study we showed that IL-27 suppressed intraocular inflammation through activation of STAT1 [21], [22]. In this study, we have investigated whether IL-27 can up-regulate the expression of CFH in retinal cells. We show here that IL-27 induces CFH expression in freshly isolated primary mouse retinal cells and human retinal epithelial cells through activation of STAT1 and up-regulation of the STAT1 target genes, Interferon Regulatory Factor-1 (IRF-1) and IRF-8.

## Methods

### Mice

C57BL/6 mice (6–8 weeks old) were from Jackson Laboratory (Bar Harbor, ME). STAT1-deficient mice were a gift from Dr D. Levy New York University NY. We generated STAT3 conditional deletion in the retina by breeding STAT3^F/F^ mice with a-Cre transgenic mice, which express Cre-recombinase only in the retina (generously provided by Dr. Gruss) [23]. Generation and characterization of transgenic mice with targeted over-expression of SOCS1 in the retina (SOCS1-Tg) has previously been described [24]. All mice were housed in clean animal rooms operated under barrier conditions. Animal care and use was in compliance with Association for Assessment and Accreditation of Laboratory Animal Care, International (AAALAC) and the NIH Intramural Animal Care & Use Program guidelines. This study was approved by the National Eye Institute/NIH Animal Care and Use Committee under the Animal Study Proposal (ASP) # NEI-597 approved on October 25, 2010).

### Induction of EAU and Imaging Mouse Fundus

We induced EAU by active immunization with bovine interphotoreceptor retinoid-binding protein (IRBP, 150 µg for C57BL/6 mice, 50 µg for B10.A mice) and human IRBP peptide (amino acid residues 1–20; 150 µg for C57BL/6 mice), in a 0.2 ml emulsion (1∶1) v/v with complete Freund’s adjuvant (CFA) containing Mycobacterium tuberculosis strain H37Ra (2.5 mg/ml). Mice also received *Bordetella pertussis* toxin (0.2 µg/mouse) concurrent with immunization. Clinical disease was established and scored by funduscopy as described [24], [25]. Briefly, following ip injection of ketamine (1.4 mg/mouse) and xylazine (0.12 mg/mouse), pupils were dilated by topical administration of 1% tropicamide ophthalmic solution (Alcon Inc, Fort Worth, Texas). To avoid a subjective bias, evaluation of the fundus photographs was conducted without knowledge of the mouse identity by a masked observer. At least six images (2 posterior central retinal view, 4 peripheral retinal views) were taken from each eye by positioning the endoscope and viewing from superior, inferior, lateral and medial fields and each individual lesion was identified, mapped and recorded. The clinical grading system for retinal inflammation was as previously established [26].

### Isolation of Retinal Cells

Primary retinal cells were isolated as previously described [22]. Briefly, retinas were dissected out from enucleated eyes and briefly rinsed in sterile PBS. Retinas were then diced in small pieces and incubated in Hanks balanced salt solution (HBSS), containing activated papain 120 U (Worthington, Lakewood NJ) and DNase 2000 U (Worthington), for 1 hour. To make single cell suspension retinas were gently triturated with pipette and then passed through a 40 µm cell strainer and centrifuged at 200 g for 5 min. The pellet was resuspended in 1% ovalbumin (Worthington) in HBSS containing 500 U of DNase and layered on 5% ovalbumin solution in HBSS and centrifuged for 10 min at 500×g. The pellet was resuspended in medium [1∶1] mixture of Dulbecco’s modified Eagle’s medium and F12 medium containing 2.5% heat-inactivated horse serum, B27 medium supplement (Invitrogen, Carlsbad, CA), 5 mg/ml D-glucose, 2 mM L-glutamine, 20 mM HEPES, 2.5 mg/ml bovine insulin (Sigma, ST Louis, MO), and 0.1 mg/ml human transferrin (Sigma; 15 000 cells/ml)]. Cell viability was consistently >90% as assessed by Trypan blue exclusion and the Vi-CELL Cell Viability Analyzer (Beckman Coulter).

### Cell Culture and Interleukin 27 (IL-27) Treatment

The human RPE cell line (ARPE-19) was purchased from the American Type Culture Collection (Manannas, VA). The ARPE-19 cells were grown to confluence in minimum essential medium (MEM) supplemented with 10% fetal bovine serum (FBS), 100 U/ml penicillin, 100 µg/ml streptomycin, and 50 ng/ml amphotericin B. The cells were washed twice in serum-free medium (SFM) (HyClone, Logan, UT) and incubated in SFM containing human recombinant IL-27 (10 ng/ml) (R&D) for varying amounts of time at 37°C in 5% CO2. The protein synthesis inhibitor, cycloheximide (CHX) (Sigma, St. Louis, MO), was used at 35 µg/ml and added 30 minutes before addition of IL-27. Treatment with CHX allows for discrimination between immediate effects of STAT1 and effects requiring de novo protein synthesis.

### Small Interfering RNA (siRNA)–mediated Silencing of CFH Expression

Cells were cultured to 60–70% confluence and transfected with siRNA oligonucleotides (Santa Cruz). Cells were replated in six-well plates and stimulated with IL-27 for varying amounts of time. Complete knockdown of CFH occurred at 72 hour-time point and was confirmed by RT-PCR analysis.

### Western Blot Analysis

Whole cell lysates were prepared as previously described [27]. Briefly, extracts were fractionated on a 4–12% gradient SDS-PAGE and proteins were then transferred to a PVDF membrane. Antibodies used were: STAT1, pSTAT1, STAT3, pSTAT3 (Cell signaling Technology), CFH, IRF-1, IRF-8, (Santa Cruz Biotechnology). Pre-immune serum was used in parallel as controls and signals were detected with HRP conjugated-secondary F(ab')2 Abs (Zymed Labs, San Francisco, CA) using the ECL Plus Kit (Amersham, Arlington Heights, IL).

### Immunohistochemistry

Enucleated eyes were fixed in 4% paraformaldehyde and embedded in Ameraffin tissue embedding medium (Baxter). Tissue sections (5 mm) were deparaffinized in xylene, rehydrated through a graded alcohol series. For detection of IRF-8, immunostaining was performed by the avidin-biotin-peroxidase complex method (Vector Laboratories, Burlingame, CA.). Sections were pre-incubation for 2 h with 5% blocking serum and then incubated overnight at 4°C with antibodies (0.02 mg/ml) specific to mouse IRF-8 (Santa Cruz). Control sections received the appropriate normal serum. All sections were subsequently incubated with biotinylated secondary antibody for 30 min at room temperature, and signal was visualized with diaminobenzidine-H_2_O_2_ as recommended (Vector). Sections were counterstained with hematoxylin. For detection of CFH expression, sections were blocked with 5% normal goat serum and then incubated with CFH antibody (Santa Cruz Biotechnology) overnight at 4°C. After washing with ICC Buffer (PBS containing 0.5% BSA, 0.2%, Tween 20 and 0.05% sodium Azide) sections were incubated with donkey anti-goat Alexa-Fluor© 488 and DAPI for 1 hr and washed with ICC Buffer. Slides were mounted with Vectashield Mounting medium for fluorescence (Vector Labs) and images taken with a confocal microscope. Gain/offset settings were set at the same level for all slides.

### Quantitative and Semi-quantitative PCR Analyses

Total RNA was extracted from primary retinal cells or ARPE-19 cells using the TriZol reagent according to the procedures recommended by the manufacturer (Life Technologies, Gaithersburg, MD). All RNA samples were digested with RNase-free DNase 1 (Life Technologies) for 30 min, purified by phenol/chloroform extractions and precipitated in 0.4 M LiCl. RNA (10 µg), SuperScript III Reverse Transcriptase (Life Technologies, Gaithersburg, MD), and oligo (dT)_12–18_ were used for first-strand synthesis as previously described [27]. Samples were subjected to hot-start RT-PCR with gene-specific primers and AmpliTaq Gold DNA polymerase (Applied Biosystems, Foster City, CA). First-strand synthesis containing each mRNA sample but no reverse transcriptase was performed to control for possible DNA contamination of mRNAs used as target for PCR amplification; failure to obtain RT-PCR products with any of the PCR amplimers confirmed the absence of contaminating DNA templates. PCR-amplified fragments were fractionated on agarose gels. All cDNA preparations used were suitable substrates for PCR amplification on the basis of efficient amplification of a ß-actin sequence. Real-time PCR was performed on an ABI 7500 and PCR parameters were as recommended for the TaqMan Universal PCR kit (Applied Biosystems). Primers and probes were purchased from Applied Biosystems).

### Statistical Analysis

Statistical analyses were performed by independent two-tailed Student’s t test. Probability values of ≤0.05 were considered statistically significant.

## Results

### IL-27 Up-regulates the Expression of CFH in Retinal Cells

**Figure 1:**
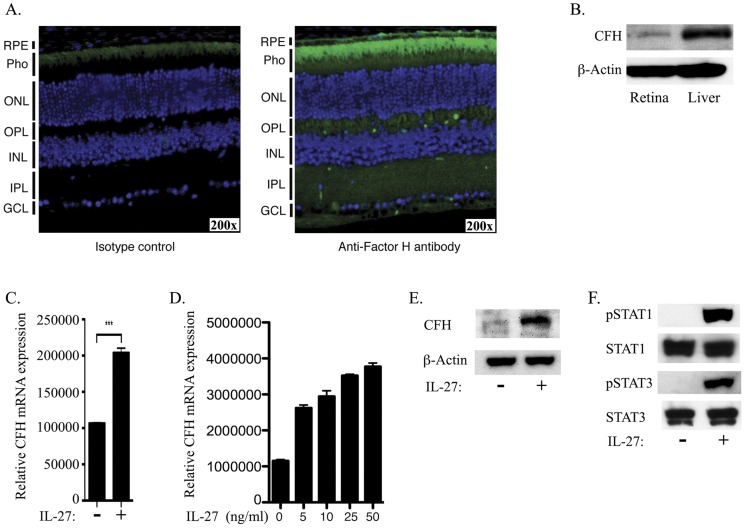
CFH is constitutively expressed in the retina and up-regulated in RPE cells by IL-27 through STAT1-dependent mechanism. (A) Immunohistochemical localization of CFH expression in the mouse retina. Retina pigmented epithelium (RPE); Photoreceptors (pho); outer nuclear layer (ONL); outer plexiform layer (OPL); inner plexiform layer (IPL). (B) Whole cell extracts prepared from the retina or liver of C57BL/6 mice were analyzed for expression of CFH by Western blot assay. (C) ARPE-19 cells were stimulated for 24 hours in medium containing human IL-27 (10 ng/ml). ARPE-19 cells were cultured overnight in complete medium, washed and starved in serum free medium (SFM) for 2 hours and then stimulated in SFM containing human recombinant IL-27 (10 ng/ml) for 24 hours (C, D, E) or 30 minutes (F). (C) RNA was isolated and analyzed by qRT-PCR. (D) The ARPE-19 cells were stimulated with several concentrations of human IL-27 and then subjected to qRT-PCR analysis. (E, F) Whole cell extracts were analyzed by Western blot assay.

CFH expression has been detected in the retina, ciliary body, sclera and the ocular lens [13]. To confirm these findings, we performed immunohistochemical analysis of 8 weeks old C57BL/6 mouse eyes and the highest level of CFH expression was detected in the RPE layer, photoreceptors, plexiform and ganglion cell layers (Fig. 1A). We also confirmed the expression of CFH in the retina of the C57BL/6 mouse by Western blot analysis. However the level expressed by retinal cells was substantially lower than the level detected in the liver (Fig. 1B). To investigate whether IL-27 induces CFH expression by the RPE, we cultured ARPE-19 cells in medium containing IL-27 and RNA analysis of CFH expression by quantitative PCR (qRT-PCR) revealed that ARPE-19 cells constitutively expressed CFH and that IL-27 significantly up-regulated the transcription of CFH (Fig. 1C) in a dose-dependent manner (Fig. 1D). These results were confirmed at the protein level by Western blotting analysis showing substantial induction of CFH protein expression by IL-27 (Fig. 1E). We further show that IL-27 induced the increase of CFH expression in ARPE-19 through activation of STAT1 and STAT3 pathways (Fig. 1F).

### CFH Expression in the Mouse Retina Requires STAT1 and is Up-regulated by IL-27

**Figure 2:**
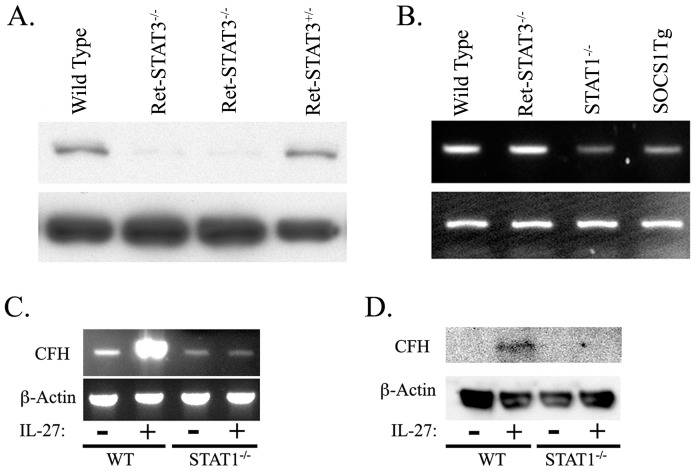
IL-27 induces CFH expression in the retina through STAT1-dependent mechanism. (A) Characterization of retina from mice with specific deletion of STAT3 in the retina (Ret-STAT3^−/−^). (B) Freshly isolated retinas from wild type (WT), STAT1 knockout (STAT1^−/−^), (Ret-STAT3^−/−^), or from mice with targeted over-expression of SOCS1 in the retina (SOCS1Tg) were analyzed by RT-PCR. (C, D) Freshly isolated retinal cells from WT or STAT1^−/−^ mice were cultured overnight, washed, starved for 2 hours in serum-free medium and then cultured for an additional 22 hours in medium containing IL-27 (10 ng/ml). We analyzed RNA and total cell extracts from the cells by RT-PCR (C) or Western blotting (D).

IL-27 induced the expression of CFH in ARPE-19 cells through the activation of both STAT1 and STAT3 (Fig. 1E, 1F). It was therefore of interest to determine whether both STAT1 and STAT3 are required for IL-27-mediated increase of CFH by analyzing CFH levels in retina of STAT1- or STAT3-deficient mice. However, because STAT3 deficiency is embryonic lethal, we used a Cre-loxP system to generate STAT3 conditional knockout mice in which STAT3 gene was disrupted only in the retina. Cre-recombinase expression was directed under control of a retina-specific regulatory element of murine Pax6 (a-Cre) [23]. Western blot analysis of whole cell extracts of the retina-STAT3 knockout mouse strain (Ret-STAT3^−/−^) indicates markedly decreased STAT3 in the retina (Fig. 2A). To address the role of STAT1 and/or STAT3 in IL-27-mediated induction of CFH, we analyzed the expression of CFH in the retina of wild type C57BL/6 (WT), STAT1-deficient (STAT1^−/−^) [28] mice or mice with targeted over-expression of SOCS1 in the retina under direction of opsin promoter (SOCS1-Tg) [24]. Western blot analysis of whole cell extracts prepared from freshly isolated retinal cells revealed equivalent levels of CFH expression in the retinas of the WT and STAT3-deficient mice (Fig. 2B), indicating that STAT3 expression and/or activation is dispensable for the expression of CFH in the retina. In contrast, we observed substantial decrease in CFH levels in the retina of the STAT1^−/−^ and SOCS1-Tg mice (Fig. 2B). It is of note that the SOCS1-Tg mice are deficient in STAT1 activation [24], underscoring the requirement of STAT1 for optimal expression of CFH in the normal retina. To confirm that STAT1 is indeed required for IL-27-mediated induction of CFH gene expression in the retina, we established retinal organ cultures from freshly isolate retinas of WT or STAT1-deficient mice and stimulated them overnight in medium containing IL-27. Results of RT-PCR analysis of the RNA (Fig. 2C) or Western blot analysis of whole cell extracts derived from the retinal cells (Fig. 2D) revealed marked increase in CFH expression by WT retinal cells cultured in medium containing IL-27. On the other hand, addition of IL-27 to STAT1-deficient retina cultures could not induce the up-regulation of CFH expression. Together, these results confirm the requirement of STAT1 for IL-27-mediated induction of CFH in the retina and underscore the physiological relevance of IL-27 in promoting the expression of CFH in the mammalian retina.

### IRF-1 and IRF-8 Contribute to Transcriptional Activation of CFH Gene in the Retina

**Figure 3:**
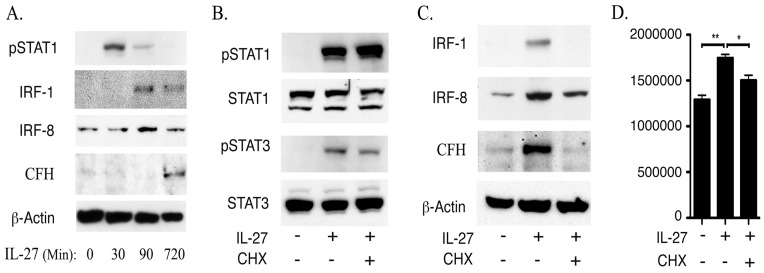
IL-27 induces expression of CFH through STAT1-mediated up-regulation of IRF-1 and IRF-8. (A–C) ARPE-19 cells were starved for 2 hours in serum free medium and cultures were then stimulated with IL-27 (10 ng/ml) for 30, 90 or 720 min. (B–D) In other cultures, cells were treated with CHX (35 ug/ml) for 30 min prior treatment with IL-27 for 30 min (B) or 24 h (C, D). Whole cell extracts were analyzed by Western blot assay and the antibodies used are indicated. (D) CFH mRNA transcripts were detected and quantified by qRT-PCR.

**Figure 4:**
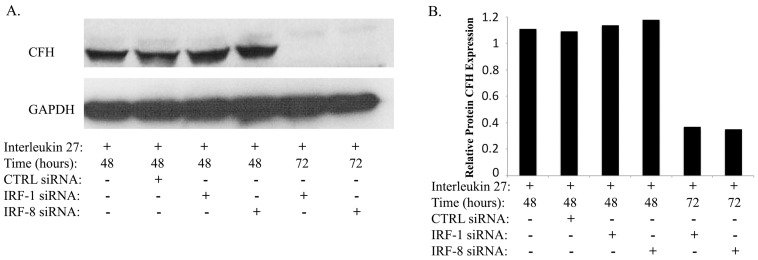
siRNA-mediated silencing of IRF-1 or IRF-8 abrogated the induction of CFH by IL-27. ARPE-19 cells were stimulated with human IL-27 in the presence or absence of control siRNA, IRF-1 siRNA or IRF-8 siRNA. (A) Detection of CFH expression by Western blot analysis. (B) Blots were analyzed and relative levels of CFH protein were quantified by densitometry; samples were normalized to the GAPDH expression level.

Interferon Regulatory Factor (IRF) family of transcription factors play important roles in gene-regulatory networks that control cell growth, differentiation and host immunity [29]. IRF-1 and IRF-8 transcription factors are induced by IFN-g through activation of STAT1 and in a previous study we showed that IFN-g signaling in human RPE is mediated by STAT1, IRF-1 and IRF-8 [30]. We therefore investigated whether the up-regulation of CFH in the retina or ARPE-19 cells by IL-27 derived from activation of *IRF-1*, *IRF-8* and/or *STAT1* genes. We starved ARPE-19 cells for 2 hours in SFM, treated the cells with IL-27 for varying amounts of time (30 minutes to 24 hours) and examined time-dependent effects of IL-27 on STAT1 activation and the expression of CFH, IRF-1 or IRF-8. Western blot analysis revealed that pSTAT1 was no longer detectable by the inception of CFH expression (Fig. 3A), suggesting that the activation of STAT1 pathway precedes CFH expression. On the other hand, the kinetics of IL-27-mediated induction of CFH protein expression correlated temporally with the expression IRF-1 and IRF-8 expression (Fig. 3A). To further discern the roles of STAT1, IRF-1 and IRF-8 in the induction of CFH expression, we blocked *de novo* protein synthesis in some cultures by adding the protein synthesis inhibitor, cycloheximide (CHX). This allowed discrimination between the immediate effects of STAT1 activation on CFH expression from secondary effects deriving from STAT1-induced expression of IRF-1 and IRF-8. The stimulation of the ARPE-19 cells with IL-27 induced robust activation of STAT1 and STAT3 pathways (pSTAT1 and pSTAT3) and was unaffected by the blockade of protein synthesis (Fig. 3B). Western blot analysis also revealed that the induction of CFH expression by IL-27 correlated temporally with the expression of IRF-1 and IRF-8 (Fig. 3C). However, blocking protein synthesis with CHX abrogated the expression of IRF-1 and significantly reduced IL-27 induction of CFH expression but had no effect on IRF-8 level (Fig. 3C). qPCR analysis revealed significant suppression of CFH mRNA transcription by cells treated with CHX despite robust STAT1 activation, indicating that de novo synthesis of IRF-1 may be required for optimal expression of *CFH* (Fig. 3D). Interestingly, the expression of CFH RNA in the CHX treated cells was still higher than untreated cells (Fig. 3D), suggesting that this increase in CFH mRNA derived from the IRF-8 still present in these cells (see Fig. 3C). To directly investigate the role of IRF-1 and IRF-8 in IL-27-mediated induction of CFH protein expression, we stimulated ARPE-19 cells with IL-27 for varying amounts of time in the presence or absence of control siRNA, IRF-1 siRNA or IRF-8 siRNA. Complete knockdown of IRF-1 or IRF-8 was observed at 72 hours and confirmed by RT-PCR analysis (data not shown). As shown siRNA-mediated knockdown of IRF-1 or IRF-8 abrogated CFH protein expression, indicating the involvement of IRF-1 and IRF-8 in CFH expression in ARPE-19 cells (Fig. 4).

### IRF-8/IRF-1 are Constitutively Expressed in Retina and Up-regulated during Ocular Inflammation

**Figure 5:**
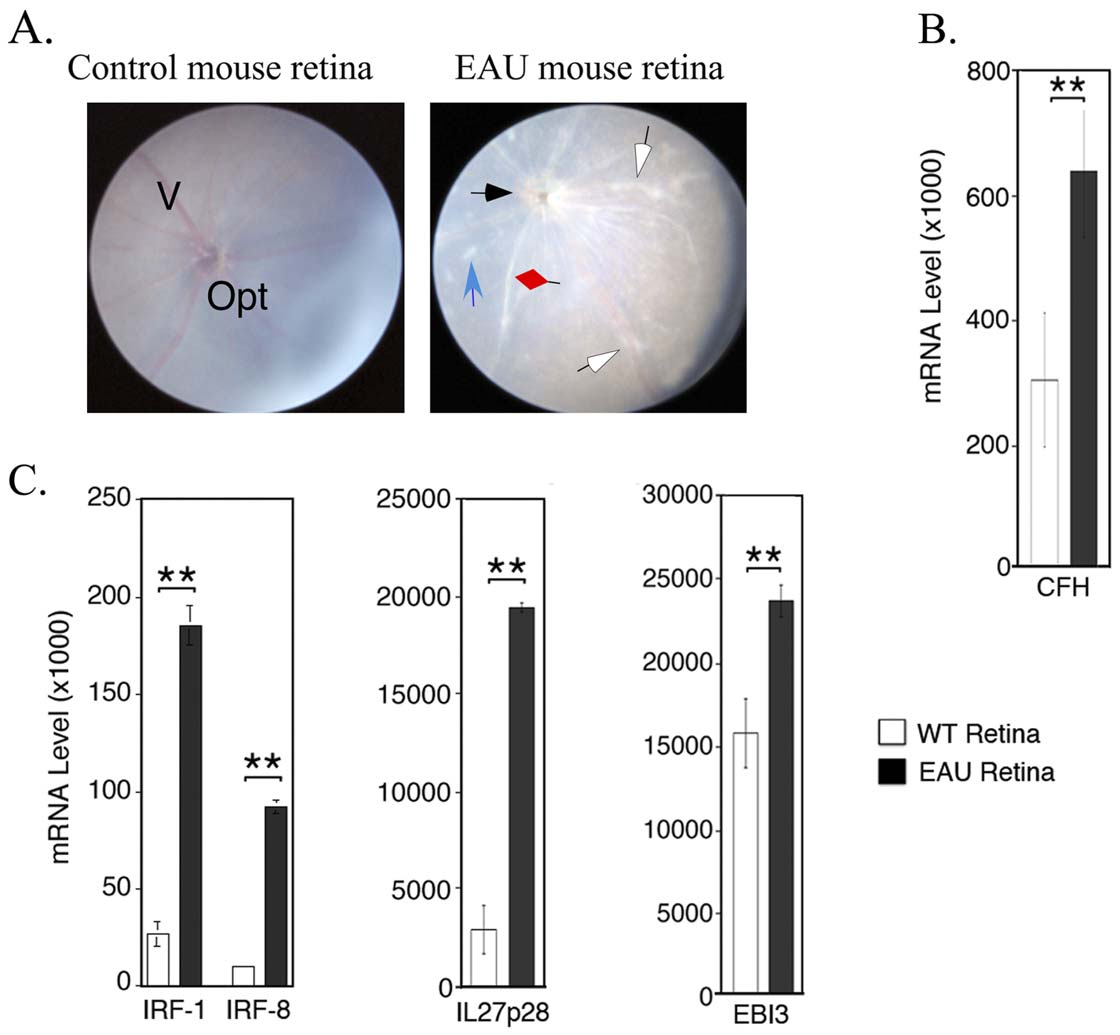
Expression of IRF-1, IRF-8 and IL-27 (IL27p28/EBi3) by retinal cells was up-regulated during experimental autoimmune uveitis (EAU). (A) Fundus images of the retina of un-immunized mouse and mouse with EAU. (B, C) Retinas were isolated from control or EAU mice and analyzed for IRF-1, IRF-8, CFH, IL27p28 or EBi3 expression by qRT-PCR.

Data presented thus far suggest the involvement of IRF-1 and IRF-8 in the regulation of CFH expression in ARPE-19 cells. It was therefore of interest to investigate whether IRF-1 and IRF-8 are expressed in the mouse retina and if their levels increase during intraocular inflammation. We addressed these points in experimental autoimmune uveitis (EAU), a mouse model of intraocular inflammation or uveitis [31], [32]. We induced EAU in C57BL/6 mice by immunization with the retinal autoantigen, interphotoreceptor retinoid binding protein (IRBP) [32], [33]. Fundus photographs showing papillitis (black arrow), retinal vasculitis (red diamond) with cuffing (blue arrows) and brownish/whitish retinal and choroidal infiltrates (white arrows) taken at day-21 post-immunization confirmed the development of EAU (Fig. 5). Retina was isolated from un-immunized or IRBP-immunized mice and RNA analysis by qRT-PCR indicates that the increase in CFH expression in the retina during inflammation (Fig. 5B) was accompanied by a corresponding increase in the transcription of genes coding for *IRF-1, IRF-8* and IL-27 (*IL27p28* and *EBI3*) (Fig. 5C).

### Detection of IRF-8 Protein in the Retina in the Ganglion and Inner Nuclear Layers of the Retina

**Figure 6:**
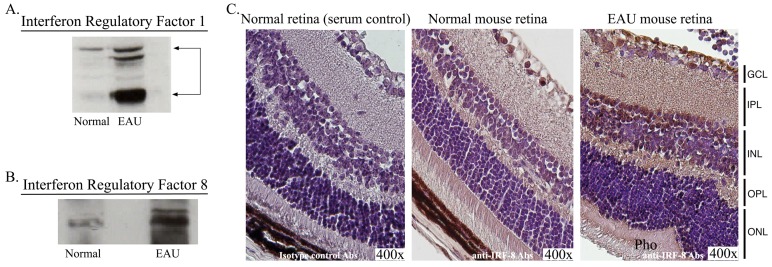
Detection and localization of IRF-1 and IRF-8 proteins in the retina. Retinas were isolated from control or EAU mice and whole cell extracts were analyzed for IRF-1 (A) or IRF-8 expression by Western blot analysis. (C) Immunohistochemical localization of IRF-8 expression was detected in the normal mouse retina or retina of mice with EAU using an IRF-8-specific antibody as described in Methods section. Retina pigmented epithelium (RPE); Photoreceptors (pho); outer nuclear layer (ONL); outer plexiform layer (OPL); inner plexiform layer (IPL).

In contrast to other members of the IRF family, IRF-8 expression has been detected mainly in myeloid cells and cells in the ocular lens [29], [34]. Although IRF-1 and IRF-8 mRNA transcripts were detected in the normal retina and up-regulated during EAU (Fig. 5C), it was important to demonstrate that the proteins were also present in the retina. Consistent with our RNA data, Western blot analysis revealed that IRF-1 and IRF-8 proteins are constitutively expressed in the normal mouse retina and their levels increased dramatically during EAU (Fig. 6A, 6B). Because cells of the myeloid/hematopoietic lineage such as microglia are resident cells of the mammalian retina [22], [35], [36], we examined whether these cells are the source of IRF-8 detected in the retina. Immunohistochememical analysis of paraffin-mixed sections using IRF-8-specific Abs detected intense immuno-reactivity within the ganglion cell layer of normal mouse retina (Fig. 6C). Consistent with increase of IRF-8 mRNA and protein in the retina during EAU, we observed marked elevation of the level of IRF-8 in ganglion and outer nuclear layers of mice with EAU (Fig. 6; right panel). Unfortunately, similar analysis using IRF-1 Abs from several commercial sources gave un-reproducible results.

## Discussion

The complement system is a critical component of innate immunity in the eye and CFH is constitutively expressed by retinal cells to protect terminally differentiated photoreceptors and retinal neurons from prolonged or inappropriate activation of the complement system in the neuroretina. Although recent studies using the ARPE-19 cell line suggest that IFN-g regulates CFH expression in RPE cells through activation of STAT1 [12], [19], factors that activate the STAT1 required for *CFH* transcription in the retina are unknown since retinal cells do not produce the pro-inflammatory cytokine, IFN-g.

In this study, we have shown that STAT1-deficient mice express much lower level of CFH in the retina compared to WT mice (Fig. 2B), suggesting that STAT1 is not only essential for activation of the transcription of *CFH* gene in ARPE-19 cells but also by resident cells of the mammalian retina. We further show that the anti-inflammatory cytokine, IL-27, induces the expression of CFH in retina (Fig. 2C, 2D) and ARPE-19 cells (Fig. 1C, 1D, 1E) in a dose dependent manner. Whereas IL-27 induced CFH in WT retinal cells, it could not up-regulate CFH expression in STAT1-deficient retinal cells indicating a requirement of STAT1-dependent mechanisms. In this context, it is interesting that STAT1 directly interacts with the IL-27p28 promoter and induces production of IL-27 by RPE cells, indicating the central role of STAT1 in regulating CFH and IL-27 [21], [22]. The requirement of STAT1 for expression of CFH and IL-27 suggests that production of the two proteins in the retina may be linked or coordinately regulated. The physiological relevance of IL-27 in the retina and RPE is further underscored by recent reports showing that the RPE, photoreceptors and retinal ganglion cells expression IL-27 as well as IL-27 receptors [21], [22]. It is particularly intriguing that the RPE and these layers of the retina express high levels of CFH [13]. Taken together, the constitutive production of IL-27 in the retina, its signaling through STAT1 and its up-regulation of CFH in ARPE-19 and mouse retinal cells, make a compelling case that IL-27 may play important roles as a physiological inducer of CFH in the mouse retina.

Blocking protein synthesis with CHX did not inhibit STAT1 activation but abrogated the IL-27-induced expression of CFH (Fig. 3C), suggesting that *de novo* protein synthesis is required for IL-27-mediated increase in CFH expression. Although previous studies have emphasized the role of STAT1 in CFH expression, our results suggest that while STAT1 is clearly required for optimal expression of CFH (Fig. 2), its role may merely be to induce expression of additional transcriptional factors that may be the direct inducers of *CFH* transcription. We have shown that the induction of CFH expression by IL-27 was dose dependent and temporally correlated with the expression of IRF-1 and IRF-8 (Fig. 3A, 3C). In addition, both IRF-1 and IRF-8 are expressed in ARPE-19 cells and mouse retina, suggesting that they may play a role in maintaining physiological levels of CFH in the retina. IRF-1 is the best characterized of the 10-member IRF family and is thought to regulate immunological responses [29], [37], [38]. Constitutive expression of IRF-1 by resident retinal cells is not particularly surprising in view of the fact that IRF-1 is ubiquitously expressed in many tissues and cell types. On the other hand, with the exception of the ocular lens [34], [39], the expression of IRF-8 has thus far been detected in cells of hematopoietic lineage [40], [41], [42]. During EAU, expression of IRF-8 and IRF-1 was dramatically increased (Fig. 6A, 6B) and we show that IRF-8 is marked elevated in ganglion and outer nuclear layers (Fig. 6C). Nonetheless, while our data indicate that IL-27 induces expression of IRF-1 and IRF-8 in the retina, a recent report indicates that IL-27p28 expression is decreased in IRF-8 knockout mice [43]. Moreover, over-expression of both IRF-8 and IRF-1 cooperatively activated IL-27p28 promoter [43], suggesting a positive regulatory mechanism for increasing IL-27, IRF-1 and IRF-8. It is therefore tempting to speculate that existence of such a feed-forward loop would undoubtedly contribute to mechanisms that sustain requisite levels of CFH in the retina.

In summary, the data presented describe the role of three transcription factors (STAT1, IRF-1, IRF-8) that converge to induce and/or enhance the expression of CFH in the retina and we have shown that they are all induced by the anti-inflammatory cytokine, IL-27. We further show that while STAT1 is required for optimal expression of CFH, its role appears to be for inducing IRF-1 and IRF-8 or other transcription factors that enhance the expression of CFH in RPE and retina. While the temporal patterns of expression suggest that IRF-1 and IRF-8 may be required for increasing CFH expression in response to IL-27 signaling, the constitutive expression of IRF-8 and IL-27 in the retina is consistent with their involvement in expression of CFH in the retina under normal physiological condition. Collectively, our data showing that IL-27 induced expression of CFH by retinal cells extends its anti-inflammatory functions in the retina and suggest that IL-27 might contribute to mechanisms of ocular immune-privilege by mitigating deleterious effects of complement proteins in retina during inflammation through activation of STAT1 and up-regulation of IRF-1 and IRF-8. Thus, it is conceivable that defects in IL-27 signaling or regulation of IRF-1/IRF-8 expression may contribute to the reduced amounts of CFH observed in retina of patients with AMD.
